# Supplementary material for: Removing the societal and legal impediments to the HIV response: An evidence-based framework for 2025 and beyond
Source: PLoS One. 2022 Feb 22;17(2):e0264249. doi: 10.1371/journal.pone.0264249 (PMC8863250; doi:10.1371/journal.pone.0264249)
Supplement: S1 Table — (DOCX) [file pone.0264249.s002.docx]

S1 Table. Search strategy

| **Database** | **Search string** | **N of records** |
| --- | --- | --- |
| **Stigma and discrimination** | | |
| Pubmed | Search: (((((Stigma[Title/Abstract]) OR (discrimination[Title/Abstract])) OR (Prejudice[MeSH Terms])) OR (stereotyping[MeSH Terms])) AND ((((((((Polic*[Title/Abstract]) OR (Healthcare workers[Title/Abstract])) OR (Sex Workers[Title/Abstract])) OR (PWID[Title/Abstract])) OR (PLHIV[Title/Abstract])) OR (LGBTQ[Title/Abstract])) OR (Youths[Title/Abstract])) OR (Adults[MeSH Terms])))) AND (((((((((((preven*[Title/Abstract]) OR (ART[MeSH Terms])) OR (Antiretroviral treatment[MeSH Terms])) OR (Linkage*[Title/Abstract]))) OR (hiv prevalence[MeSH Terms])) OR (hiv infection*[MeSH Terms])))) OR (HIV/AIDS[Title/Abstract])) OR (adherence[Title/Abstract])) | 8881 |
| Scopus | TITLE-ABS ( stigma ) OR TITLE-ABS ( discrimination ) OR TITLE-ABS ( prejudice ) OR TITLE-ABS ( stereotyping ) AND TITLE-ABS ( polic* ) OR TITLE-ABS ( "Healthcare workers" ) OR TITLE-ABS ( "Sex Workers" ) OR TITLE-ABS ( pwid ) OR TITLE-ABS ( plhiv ) OR TITLE-ABS ( lgbtq ) OR TITLE-ABS ( youths ) OR TITLE-ABS ( adults ) AND TITLE-ABS ( preven* ) OR TITLE-ABS ( art ) OR TITLE-ABS ( "Antiretroviral treatment" ) OR TITLE-ABS ( linkage ) OR TITLE-ABS ( "HIV prevalence" ) OR TITLE-ABS ( "HIV infection*" ) OR TITLE-ABS ( hiv/aids ) OR TITLE-ABS ( adherence ) | 4622 |
| Web of Science | TS=(preven* OR art OR "Antiretroviral treatment" OR linkage OR "HIV prevalence" OR "HIV infection*" OR hiv/aids OR adherence) AND TS=(polic* OR "Healthcare workers" OR "Sex Workers" OR pwid OR plhiv OR lgbtq OR youths OR adults) AND TS=(stigma OR discrimination OR prejudice OR stereotyping) | 6018 |
| **Legal environment and social justice** | | |
| Pubmed | Search: (((((((((human rights[Title/Abstract]) OR (inject drugs[Title/Abstract])) OR (structural violence[Title/Abstract])) OR (law enforcement[Title/Abstract])) OR (Decriminalizing[Title/Abstract])) OR (evaluating law[Title/Abstract])) OR (syringe[Title/Abstract])) OR (social programming[Title/Abstract])) AND ((((((((Polic*[Title/Abstract]) OR (Healthcare workers[Title/Abstract])) OR (Sex Workers[Title/Abstract])) OR (PWID[Title/Abstract])) OR (PLHIV[Title/Abstract])) OR (LGBTQ[Title/Abstract])) OR (Youths[Title/Abstract])) OR (Adults))) AND ((((((((((preven*[Title/Abstract]) OR (ART[MeSH Terms])) OR (Antiretroviral treatment[MeSH Terms])) OR (Linkage*[Title/Abstract])) OR (Viral[MeSH Terms])) OR (hiv prevalence[MeSH Terms])) OR (hiv infection*[MeSH Terms])) OR (Health Knowledge[MeSH Terms])) OR (HIV/AIDS[Title/Abstract])) OR (adherence[Title/Abstract])) | 4521 |
| Scopus | TITLE-ABS ( "human rights") OR TITLE-ABS ( "inject drugs") OR TITLE-ABS ( "structural violence") OR TITLE-ABS ( "law enforcement") OR TITLE-ABS ( Decriminalizing ) OR TITLE-ABS ( "evaluating law") OR TITLE-ABS ( syringe) OR TITLE-ABS ( "social programming") AND TITLE-ABS ( polic* ) OR TITLE-ABS ( "Healthcare workers" ) OR TITLE-ABS ( "Sex Workers" ) OR TITLE-ABS ( pwid ) OR TITLE-ABS ( plhiv ) OR TITLE-ABS ( lgbtq ) OR TITLE-ABS ( youths ) OR TITLE-ABS ( adults ) AND TITLE-ABS ( preven* ) OR TITLE-ABS ( art ) OR TITLE-ABS ( "Antiretroviral treatment" ) OR TITLE-ABS ( linkage ) OR TITLE-ABS ( "HIV prevalence" ) OR TITLE-ABS ( Viral ) OR TITLE-ABS ( "HIV infection*" ) OR TITLE-ABS ( "Health Knowledge" ) OR TITLE-ABS ( hiv/aids ) OR TITLE-ABS ( adherence ) | 4816 |
| Web of Science | TS=(preven* OR art OR "Antiretroviral treatment" OR linkage OR "HIV prevalence" OR viral OR "HIV infection*" OR hiv/aids OR adherence OR "Health Knowledge") AND TS=(polic* OR "Healthcare workers" OR "Sex Workers" OR pwid OR plhiv OR lgbtq OR youths OR adults) AND TS=("human rights" OR "inject drugs" OR "structural violence" OR "law enforcement" OR Decriminalizing OR "evaluating law" OR syringe OR "social programming") | 4321 |
| **Gender equality** | | |
| Pubmed | (((Sex Workers[Title/Abstract]) OR (PWID[Title/Abstract]) OR (PLHIV[Title/Abstract]) OR (LGBTQ[Title/Abstract]) OR "male"[Title/Abstract] OR "men"[Title/Abstract] OR "female"[Title/Abstract] OR "women"[Title/Abstract])) AND ("Gender"[Title/Abstract] OR "empowerment"[Title/Abstract] OR "condom"[Title/Abstract] OR "violence"[Title/Abstract] ) AND (("HIV prevention"[Title/Abstract]) OR ("HIV incidence"[Title/Abstract]) OR (Linkage[Title/Abstract]) OR ("Viral suppression"[Title/Abstract]) OR (hiv infection*[MeSH Terms]) OR ("ART adherence"[Title/Abstract]) OR "HIV test*[Title/Abstract]") | 12145 |
| Scopus | TITLE-ABS ( Gender ) OR TITLE-ABS ( empowerment ) OR TITLE-ABS ( condom ) OR TITLE-ABS ( violence ) AND TITLE-ABS ( "Sex Workers" ) OR TITLE-ABS ( pwid ) OR TITLE-ABS ( plhiv ) OR TITLE-ABS ( lgbtq ) OR TITLE-ABS ( women ) OR TITLE-ABS ( female ) OR TITLE-ABS ( men ) OR TITLE-ABS ( male ) AND TITLE-ABS ( "HIV prevention" ) OR TITLE-ABS ( "HIV incidence" ) OR TITLE-ABS ( Linkage ) OR TITLE-ABS ( linkage ) OR TITLE-ABS ( "Viral suppression" ) OR TITLE-ABS ( "HIV infection*" ) OR TITLE-ABS ( ART ) OR TITLE-ABS ( adherence ) OR TITLE-ABS ( "HIV test*" ) | 15132 |
| Web of Science | TS=("HIV prevention" OR "HIV incidence" OR linkage OR "viral suppression" OR "HIV infection*" OR ART OR adherence OR "HIV test*") AND TS=( "Sex Workers" OR pwid OR plhiv OR lgbtq OR women OR female OR men OR male) AND TS=(Gender OR empowerment OR condom OR violence ) | 18080 |
