# Supplementary material for: Removing the societal and legal impediments to the HIV response: An evidence-based framework for 2025 and beyond
Source: PLoS One. 2022 Feb 22;17(2):e0264249. doi: 10.1371/journal.pone.0264249 (PMC8863250; doi:10.1371/journal.pone.0264249)
Supplement: S2 Table. Study and intervention characteristics, HIV outcomes assessed, and study findings on key areas for development co-action from 16 studies — (DOCX) [file pone.0264249.s003.docx]

S2 Table. Study and intervention characteristics, HIV outcomes assessed, and study findings on key areas for development co-action from 16 studies.

| **1^st^ Author, publication date, country, study design^A^** | **Study Population^B^** | **Sample** | **Intervention/Policy Description, duration *or* Social impediment studied** | **Socio-ecological Levels** | **HIV Outcomes** | **Results**  **(Positive, Negative, No effect; Details)** |
| --- | --- | --- | --- | --- | --- | --- |
| ***Co-action across development sectors (n=16)*** | | | | | | |
| Aibibula, 2016, USA and Uganda (SR-MA) (1) | PLHIV | 4,589 PLHIV from two countries (USA and Uganda) in eight studies (three cohort and five cross-sectional studies) | No intervention; food insecurity | n/a | CD4 count | *Negative*  Food insecurity linked with 1.32 greater odds of having a low CD4 count.  Pooled OR: 1.32, 95% CI 1.15–1.53, I^2^=22.4% |
| Aibibula, 2018, Canada (O-RXS) (2) | People coinfected with HIV and hepatitis C virus | 725 people with 1,973 person-visits | No intervention; food insecurity | n/a | HIV viral load  CD4 count | *Negative*  People on treatment with severe food insecurity had 1.47 times the risk of having a detectable viral load and a 0.91-fold increase in  CD4 count  RR for viral load: 1.47, 95% CI 1.14-1.88  Fold increase in CD4 count: 0.91, 95% CI 0.84-0.98 |
| Chop, 2017 (SR) (3) | Women living with HIV | 2,492 people of which 746 were women from seven countries (Canada, the Democratic Republic of  Congo, France, Swaziland, USA, Uganda and Zambia) in seven studies (five qualitative studies, one cohort and one cross-sectional study) | No intervention; food insecurity | n/a | ART adherence, transactional sex, condom-less sex | *Negative*  Food insecurity emerged as a key reason for non-adherence to ART.  Food insecurity emerged as a key reason for engaging in transactional sex and condom-less sex. |
| Cluver, 2016, South Africa (O/XS) (4) | Adolescents living with HIV taking ART | 1,059 | Combination social protection including food security, HIV support group and good parental supervision/monitoring | Individual; interpersonal | ART adherence | *Positive*  Combination social protection showed additive benefits. With no social protection, non-adherence was 54%, with any one protection 39-41%, with any two social protections, 27-28% and with all three social protections (Food security, HIV support group and good parental supervision/monitoring), 18%.  Food security: OR=0.565, 95% CI 0.418-0.763  HIV support group: OR=0.603, 95% CI 0.401-0.906  Parental supervision/monitoring: OR=0.557, 95% CI 0.426-0.728 |
| De Neve, 2015, Botswana (QE) (5) | General population and PLHIV | 1,760 women and 1,354 men in Botswana AIDS  Impact Survey 2004  2,205 women and 1,699 men in Botswana AIDS  Impact Survey 2008 | Policy reform in secondary schooling that took place in January 1996, in which the tenth year of education shifted from senior secondary to junior secondary school. | Public Policy | HIV infection | *Positive*  The study found an 8.1% reduction in risk of HIV infection per year among boys and girls combined, and an 11.6% reduction per year for girls alone.  Effect estimate (SE) in girls: -11.6 (5.8)  Effect estimate (SE) in boys: -5.0 (2.9)  Effect estimate (SE) combined: -8.1 (3.1) |
| de Pee, 2014 (SR) (6) | PLHIV and/or TB | PLHIV from five countries (India, Niger, Zambia, Mozambique, Haiti) in six studies | Food assistance; duration varied across studies | Individual | ART adherence | *Positive*  Food assistance improves ART adherence  98 % of food assisted and 77.4 % of non-food assisted remained adherent to ART over 6-month period (p<0.05)  Food assistance recipients had higher ART adherence compared to non-recipients (98.3 vs 88.8 %, respectively; p<0.01). |
| Harris et al 2017, United States (SR-MA) (7) | People living with HIV | 10,556 PLHIV in ten studies in the US | No intervention; housing stability | n/a | ART adherence | *Positive*  The summary effect for the association between housing stability and medication adherence was positive and significant (standardized mean difference = 0.15, 95% CI: 0.02 to 0.29) - I^2^=64.1% |
| Hatcher et al. 2019 South Africa (O/XS; SEM) (8) | Peri-urban men | 2006 currently partnered men | No intervention; food insecurity | n/a | Intimate partner violence perpetration | *Negative*  Food insecurity was associated with doubled odds of intimate partner violence  OR=2.15, 95% CI=1.73, 2.66. |
| Kalichman, 2014, United States (O/XS) (9) | PLHIV | 364 men and 157 women | No intervention; food insecurity | n/a | ART adherence | *Negative*  Food insecurity was associated with poorer ART adherence, running out of ART:  <85% adherent (OR=0.43 CI=0.20, 0.92)  Runs out of ART (OR=3.22 CI=1.33, 7.18)  People without transportation were 4.4 to 6.3 times more likely to agree to key food insecurity indicators. Transportation was the only poverty risk factor that was consistently associated with food insecurity. |
| Mantsios, 2018, Tanzania (O/XS) (10) | Female sex workers | 496 | Community savings group | Community | Consistent condom use | FSWs participating in a community savings group were significantly more likely to report consistent condoms use with new clients in the last 30 days  aOR = 1.77, 95% CI 1.10-2.86 |
| Mensch, 2020, Malawi (SA) (11) | Adolescents | 2,649 young people aged 14–17 | No intervention; school enrolment, grade attainment, and academic skills—numeracy and Chichewa literacy | n/a | Herpes simplex virus type 2 (HSV-2); HIV incidence | *Positive*  Grade attainment was significantly associated with lower rates of both HSV-2 and HIV among girls, and was negatively associated with HSV-2 but not HIV among boys.  *No effect*  School enrolment and academic skills are not significantly associated with sexually transmitted infections (STIs) for boys or girls in our final models. |
| Munoz, 2011, Peru (CCS; SA) (12) | PLHIV on ART | 95 | Microfinance programme (Community-based Accompaniment with Supervised Antiretrovirals, CASA) that included transportation and nutrition support | Community | Taking ART, ART adherence, virologic suppression | At 2 years, CASA participants were more more likely to be on HAART, 86.7% versus 51.7%, X^2^ = 17.7, P < 0.01, and achieve virologic suppression, 66.7% versus 46.7%, X2 = 4.9, P = 0.03, and report adherence to HAART, 79.3% versus 44.1%, X^2^ = 15.3, P < 0.01, |
| Reed, 2010, India (O/XS) (13) | Female sex workers | 673 | No intervention;  Financial debt | n/a | Physical violence, unprotected sex with clients/partners, anal sex with clients, STI symptoms, | FSWs who reported debt were more likely to report the following:  Recent physical violence (aOR== 2.4, 95% CI: 1.5-3.9),  Unprotected sex with occasional clients in the past week (\OR=2.3, 95% CI 1.2-4.3)  Anal sex with clients in the past 30 days aOR=2.0, 95% CI 1.1-3.9)  At least one sexually transmitted infection symptom in the past six months  aOR=1.6, 95% CI 1.1-2.4).  FSWs with debt were more likely to report current husbands or other male partners, and less likely to report condom use with these partners, further increasing their sexual risk. |
| Tucker, 2017 (Qualitative SR-MS) (14) | PLHIV | 24 studies from 12 countries (Ethiopia, Tanzania, Zimbabwe, Malawi, Nigeria, Kenya, South Africa, Botswana, India, UK, US, Canada) | No intervention; poverty, housing, food insecurity, transportation, gender | n/a | ART adherence, retention, linkage | Gender had a strong influence on the uptake  of public health HIV interventions in low and middle-income African countries.  Among men living with HIV, interventions and clinic systems were perceived not responsive to their needs, contributing to poor engagement in low and middle-income African countries but not in high-income settings.  Four structural issues (poverty, unstable housing,  food insecurity, lack of transportation) mediated the feasibility and acceptability of public health HIV interventions. |
| Weinhardt, 2017, Malawi (QE) (15) |  | 899 individuals and 1002 households | Large-scale, multi-level economic and food security intervention | Individual and community | HIV testing  HIV case finding | Intervention led to increased HIV testing (OR 1.90; 95 % CI 1.29-2.78) and HIV case finding (OR = 2.13; 95 % CI 1.07-4.22) |
| Witte, 2015, Mongolia (RCT) (16) | Female sex workers | 107 | A structural intervention combining savings-led microfinance and HIV prevention components | Individual | Unprotected vaginal sex acts | Intervention was related to a higher likelihood to report zero unprotected vaginal sex acts with paying sexual partners  Zero-inflated Odds and 95% CI: 3.72 (–0.37, 7.80) |

^A^ Study design abbreviations: B/A: Before/after study; CRT: Cluster randomised trial; CCS: Case-control study; MM = mixed methods; MS: Modelling study; O/XS = observational cross-section; O/RXS = observational repeated cross-sections; PR = policy review; PMD = program monitoring data; RCT: Randomised controlled trial; QE = Quasi-experimental study design; QP = qualitative post-test only; SR: Systematic review; SR-MA: Systematic review with meta-analysis; SR-MS: Systematic review with meta-synthesis; SEM: Structural equation modeling; SA: Survival analysis

^B^ HCW= healthcare workers; LGBTQ = lesbian, gay, bisexual, transgender, and questioning; MSM = men who have sex with men; PLHIV = people living with HIV; PWID = people who inject drugs; SW = sex workers;

aRR: adjusted relative risk; aOR: adjusted odds ratio; CI: Confidence intervals; OR: Odds ratio; RR: Relative Risk; SE: Standard Error; I^2^: testing the statistical heterogeneity among the studies; IRR: Incidence rate ratio

**References**

1. Aibibula W, Cox J, Hamelin AM, Mamiya H, Klein MB, Brassard P. Food insecurity and low CD4 count among HIV-infected people: a systematic review and meta-analysis. AIDS Care - Psychol Socio-Medical Asp AIDS/HIV. 2016 Dec 1;28(12):1577–85.

2. Aibibula W, Cox J, Hamelin A-M, Moodie E, Naimi A, McLinden T, et al. Food insecurity may lead to incomplete HIV viral suppression and less immune reconstitution among HIV/hepatitis C virus-coinfected people. HIV Med. 2018 Feb;19(2):123–31.

3. Chop E, Duggaraju A, Malley A, Burke V, Caldas S, Yeh PT, et al. Food insecurity, sexual risk behavior, and adherence to antiretroviral therapy among women living with HIV: A systematic review. Health Care Women Int. 2017 Sep 2;38(9):927–44.

4. Cluver LD, Toska E, Orkin FM, Meinck F, Hodes R, Yakubovich AR, et al. Achieving equity in HIV-treatment outcomes: can social protection improve adolescent ART-adherence in South Africa? AIDS Care. 2016 May 26;28:73–82.

5. De Neve J-W, Fink G, Subramanian S V, Moyo S, Bor J. Length of secondary schooling and risk of HIV infection in Botswana: evidence from a natural experiment. Lancet Glob Heal. 2015 Aug;3(8):e470–7.

6. de Pee S, Grede N, Mehra D, Bloem MW. The Enabling Effect of Food Assistance in Improving Adherence and/or Treatment Completion for Antiretroviral Therapy and Tuberculosis Treatment: A Literature Review. AIDS Behav. 2014 Oct 1;18:531–41.

7. Harris RA, Xue X, Selwyn PA. Housing stability and medication adherence among HIV-positive individuals in antiretroviral therapy: A meta-analysis of observational studies in the United States. J Acquir Immune Defic Syndr. 2017 Mar 1;74(3):309–17.

8. Hatcher AM, Stöckl H, McBride RS, Khumalo M, Christofides N. Pathways From Food Insecurity to Intimate Partner Violence Perpetration Among Peri-Urban Men in South Africa. Am J Prev Med. 2019 May 1;56(5):765–72.

9. Kalichman SC, Hernandez D, Cherry C, Kalichman MO, Washington C, Grebler T. Food Insecurity and Other Poverty Indicators Among People Living with HIV/AIDS: Effects on Treatment and Health Outcomes. J Community Health. 2014 Oct 31;39(6):1133–9.

10. Mantsios A, Galai N, Mbwambo J, Likindikoki S, Shembilu C, Mwampashi A, et al. Community Savings Groups, Financial Security, and HIV Risk Among Female Sex Workers in Iringa, Tanzania. AIDS Behav. 2018 Nov 1;22(11):3742–50.

11. Mensch BS, Grant MJ, Soler-Hampejsek E, Kelly CA, Chalasani S, Hewett PC. Does schooling protect sexual health? The association between three measures of education and STIs among adolescents in Malawi. Popul Stud (NY). 2020 May 3;74(2):241–61.

12. Muñoz M, En Salud Sucursal Perú S, Jaime Bayona P, Eduardo Sanchez P, Nacional Hipólito Unanue H, Jorge Arevalo P, et al. Matching Social Support to Individual Needs: A Community-Based Intervention to Improve HIV Treatment Adherence in a Resource-Poor Setting HHS Public Access. AIDS Behav. 2011;15(7):1454–64.

13. Reed E, Gupta J, Biradavolu M, Devireddy V, Blankenship KM. The Context of Economic Insecurity and Its Relation to Violence and Risk Factors for HIV Among Female Sex Workers in Andhra Pradesh, India. Vol. 125, Research Articles Public Health Reports. 2010.

14. Tucker JD, Tso LS, Hall B, Ma Q, Beanland R, Best J, et al. Enhancing Public Health HIV Interventions: A Qualitative Meta-Synthesis and Systematic Review of Studies to Improve Linkage to Care, Adherence, and Retention. EBioMedicine. 2017 Mar 1;17:163–71.

15. Weinhardt L, Galvao L, Yan A, Stevens P, Mwenyekonde T, Ngui E, et al. Mixed-Method Quasi-Experimental Study of Outcomes of a Large-Scale Multilevel Economic and Food Security Intervention on HIV Vulnerability in Rural Malawi. AIDS Behav. 2017;21(3):712–23.

16. Witte SS, Aira T, Tsai LC, Riedel M, Offringa R, Chang M, et al. Efficacy of a savings-led microfinance intervention to reduce sexual risk for HIV among women engaged in sex work: A randomized clinical trial. Am J Public Health. 2015 Mar 1;105(3):e95–102.
