# Supplementary material for: Removing the societal and legal impediments to the HIV response: An evidence-based framework for 2025 and beyond
Source: PLoS One. 2022 Feb 22;17(2):e0264249. doi: 10.1371/journal.pone.0264249 (PMC8863250; doi:10.1371/journal.pone.0264249)
Supplement: S3 Table. Societal enabler target for achievement by 2025 in the development sector and recommended indicators to assess progress — (DOCX) [file pone.0264249.s004.docx]

S3 Table. Societal enabler target for achievement by 2025 in the development sector and recommended indicators to assess progress

| **Top-line Targets** | **Detailed Targets** | **Recommended Indicators** | **Baseline values** |
| --- | --- | --- | --- |
| **Co-action across development sectors**  4. Achieve SDG targets critical to the HIV response (i.e., 1 – no hunger, 2-zero poverty, 3-good health and well-being, 4-quality education, 5-gender equality, 8-decent work and economic growth, 10-reduced inequalities, 11-sustainable cities and communities) by 2030 | Not applicable | SDG 1.2.1 Proportion of population living below the national poverty line, by sex and age | See <https://sdg-tracker.org/no-poverty> |
|  |  | SDG 1.3.1 Proportion of population covered by social protection floors/systems, by sex, distinguishing children, unemployed persons, older persons, persons with disabilities, pregnant women, newborns, work-injury victims and the poor and the vulnerable |  |
|  |  | SDG 1.4.1 Proportion of population living in households with access to basic services |  |
|  |  | SDG 2.1.1 Prevalence of undernourishment |  |
|  |  | SDG 4.1.2 School completion rate (lower secondary education) |  |
|  |  | SDG 4.7.2 Proportion of schools that provided life skills-based HIV and sexuality education in the previous academic year. |  |
|  |  | SDG 5.6.1 Proportion of women aged 15–49 years who make their own informed decisions regarding sexual relations, contraceptive use, and reproductive health care |  |
|  |  | SDG 5.a.1 (a) Proportion of total agricultural population with ownership or secure rights over agricultural land, by sex; and (b) share of women among owners or rights-bearers of agricultural land, by type of tenure |  |
|  |  | SDG 5.a.2 Proportion of countries where the legal framework (including customary law) guarantees women’s equal rights to land ownership and/or control |  |
|  |  | SDG 5.b.1 Proportion of individuals who own a mobile telephone, by sex |  |
|  |  | SDG 5.c.1 Proportion of countries with systems to track and make public allocations for gender equality and women’s empowerment |  |
|  |  | SDG 8.1.1 Annual growth rate of real GDP per capita |  |
|  |  | SDG 8.3.1 Proportion of informal employment in total employment, by sector and sex |  |
|  |  | SDG 10.2.1 Proportion of people living below 50 per cent of median income, by sex, age and persons with disabilities |  |
|  |  | SDG Indicator 11.1.1 Proportion of urban population living in slums, informal settlements, or inadequate housing |  |
|  |  | SDG 11.2.1Proportion of population that has convenient access to public transport, by sex, age, and persons with disabilities |  |
