# Supplementary figures and images for: Removing the societal and legal impediments to the HIV response: An evidence-based framework for 2025 and beyond
Source: PLoS One. 2022 Feb 22;17(2):e0264249. doi: 10.1371/journal.pone.0264249 (PMC8863250; doi:10.1371/journal.pone.0264249)

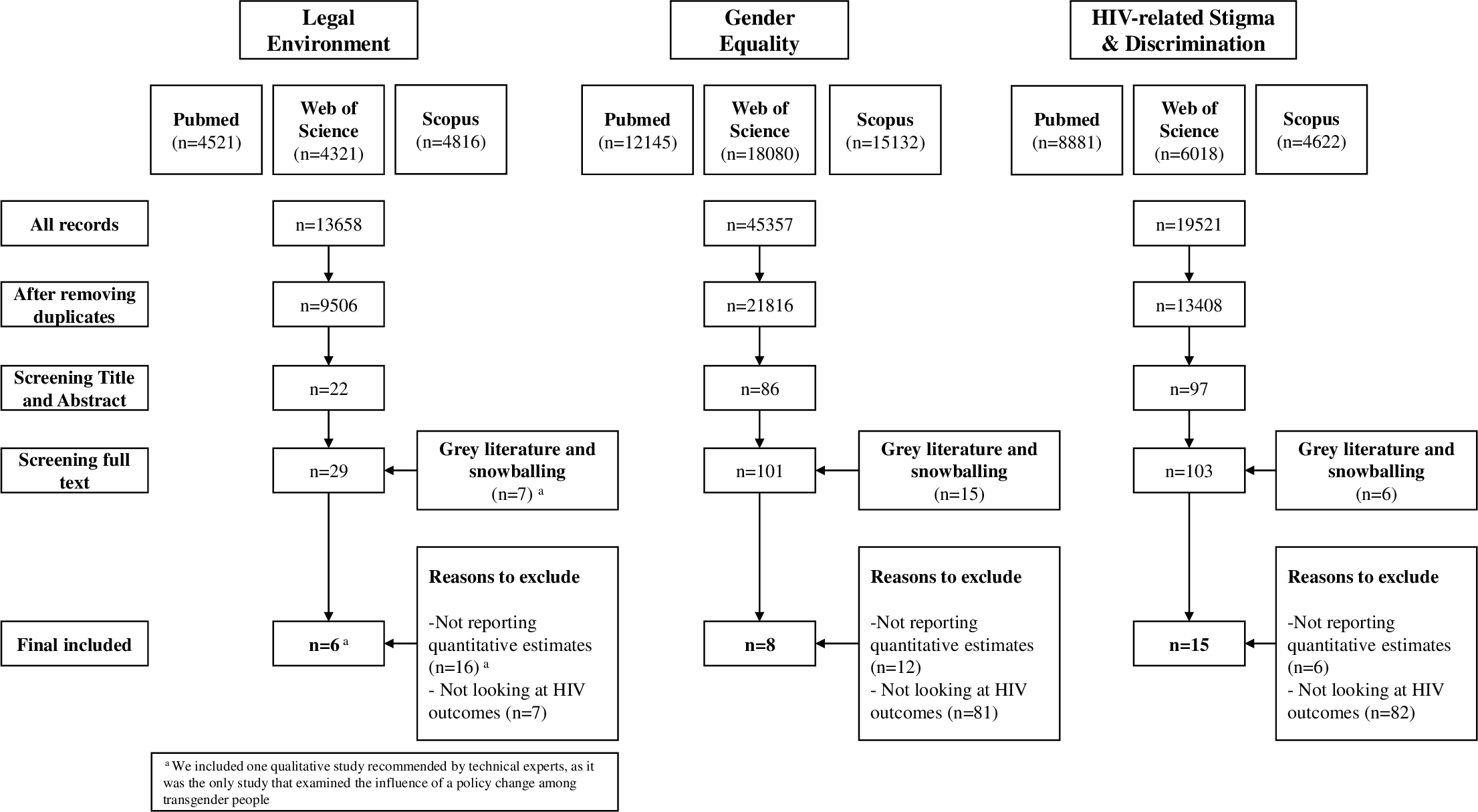

Supplement: S1 Fig — (TIF) [file pone.0264249.s005.tif]

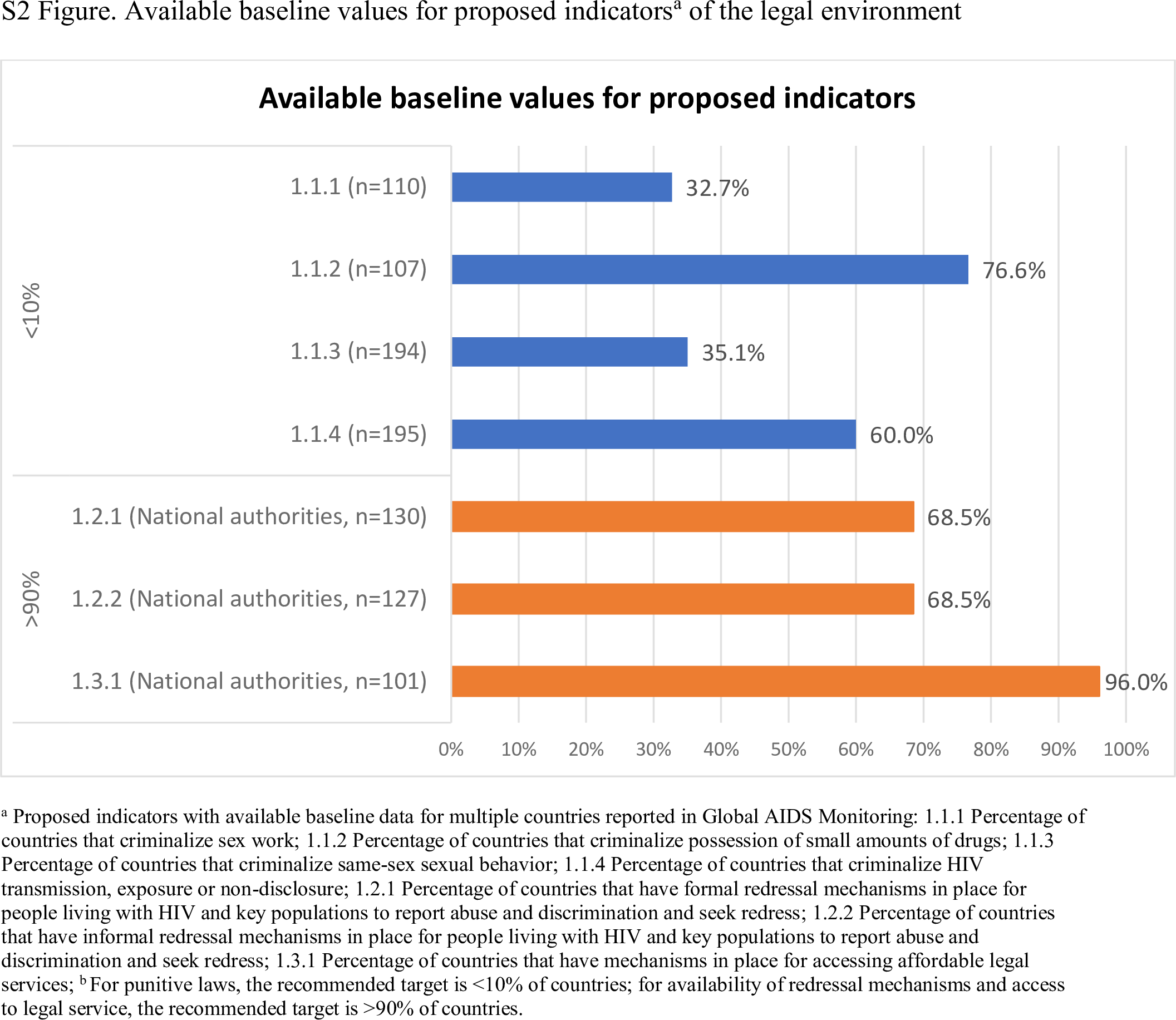

Supplement: S2 Fig — (TIF) [file pone.0264249.s006.tif]

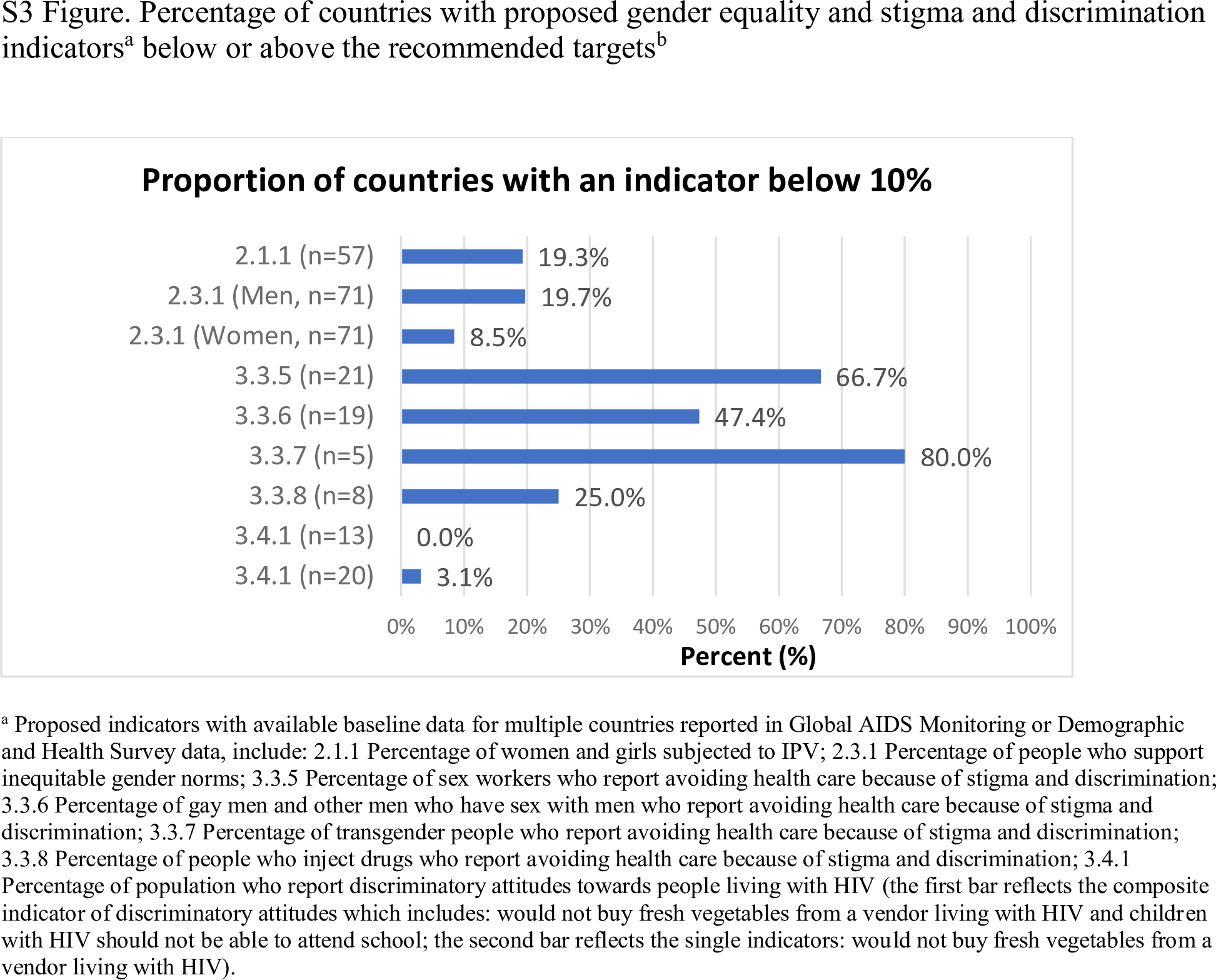

Supplement: S3 Fig — (TIF) [file pone.0264249.s007.tif]
