# Supplementary material for: Removing the societal and legal impediments to the HIV response: An evidence-based framework for 2025 and beyond
Source: PLoS One. 2022 Feb 22;17(2):e0264249. doi: 10.1371/journal.pone.0264249 (PMC8863250; doi:10.1371/journal.pone.0264249)
Supplement: Attachment — Submitted filename: Response to Reviewers.docx [file pone.0264249.s009.docx]

| **Journal Requirements** |  |
| --- | --- |
| Please ensure that your manuscript meets PLOS ONE's style requirements, including those for file naming | Done. We have reviewed the requirements for formatting of the manuscript, title page and affiliations, figures and tables and references and all have been updated to meet the requirements. |
| Please complete and upload a PRISMA-ScR Checklist | Done. We have uploaded the checklist as supplemental information. |
| a) Please clarify the sources of funding (financial or material support) for your study. List the grants or organizations that supported your study, including funding received from your institution.  b) State what role the funders took in the study. If the funders had no role in your study, please state: “The funders had no role in study design, data collection and analysis, decision to publish, or preparation of the manuscript.”  c) If any authors received a salary from any of your funders, please state which authors and which funders.  d) If you did not receive any funding for this study, please state: “The authors received no specific funding for this work.”  Please include your amended statements within your cover letter; we will change the online submission form on your behalf. | We have included the following financial disclosure on page 31-32 of the revised manuscript:  “ALS and TP received funding from the Joint United Nations Programme on HIV/AIDS (UNAIDS) for the preparation of this article. JAI-L and VB are salaried employees of UNAIDS. The funders commissioned the study. JAI-L participated in the conceptualization of the study, the preparation of the manuscript and the decision to publish.” |
| Please complete your Competing Interests on the online submission form to state any Competing Interests. If you have no competing interests, please state "The authors have declared that no competing interests exist." | We have added the following statement about competing interests on page 32 of the revised manuscript.  “The authors have declared that no competing interests exist.” |
| In your Data Availability statement, you have not specified where the minimal data set underlying the results described in your manuscript can be found. PLOS defines a study's minimal data set as the underlying data used to reach the conclusions drawn in the manuscript and any additional data required to replicate the reported study findings in their entirety. All PLOS journals require that the minimal data set be made fully available. | We have added the following data availability statement on page 32 of the revised manuscript.  “Following publication, the data reported will be accessible via the Systematic Review Data Repository (SDRH) at: [http://srdr.ahrq.gov](http://srdr.ahrq.gov/).” |
| Upon re-submitting your revised manuscript, please upload your study’s minimal underlying data set as either Supporting Information files or to a stable, public repository and include the relevant URLs, DOIs, or accession numbers within your revised cover letter. | Done. We have uploaded our underlying data set as a supplemental file. |
| We note that you have stated that you will provide repository information for your data at acceptance. Should your manuscript be accepted for publication, we will hold it until you provide the relevant accession numbers or DOIs necessary to access your data. If you wish to make changes to your Data Availability statement, please describe these changes in your cover letter and we will update your Data Availability statement to reflect the information you provide. | Noted. We plan to also upload the data to the SDSR repository prior to publication, but the website is currently down. |
| Please review your reference list to ensure that it is complete and correct. If you have cited papers that have been retracted, please include the rationale for doing so in the manuscript text or remove these references and replace them with relevant current references. Any changes to the reference list should be mentioned in the rebuttal letter that accompanies your revised manuscript. If you need to cite a retracted article, indicate the article’s retracted status in the References list and include a citation and full reference for the retraction notice. | Done. The reference list has been reviewed and updated to ensure that it is correct. We did not remove any references. We added one reference related to cost-effectiveness (reference 66). We made a few corrections to the existing references to ensure that the references were displayed correctly.  66. Institute of Medicine (US). The Best Intentions: Unintended Pregnancy and the Well-Being of Children and Families. Brown S, Eisenberg L, editors. Washington, DC: National Academies Press (US); 1995. |
| **Editor’s comments to the Author:** |  |
| I especially agree with the reviewer's comment regarding the need for a specific, but brief, discussion delineating the differences between effectiveness and cost-effectiveness with respect to evaluation of HIV interventions. | We also agree and have added in text in response to the reviewer’s suggestion (see details below). |
| **Reviewer’s comments to the Author:** |  |
| 1. The article would benefit from a short discussion of the difference between effectiveness and cost-effectiveness in evaluating HIV interventions (line 110). We have long known that there is a positive causative relation between many of the interventions described and reduced HIV transmission / increased treatment access and adherence. Missing is cost-effectiveness data (e.g. unit cost of HIV infection averted) which would justify massive scale up of investment in these interventions when compared with other social interventions. The Global Fund invests less than 1% of grants in so-called ‘human rights programmes.’ This will only change when the data show the relative cost-effectiveness of the proposed programmes. We are waiting for these data from the Global Fund’s Breaking Down Barriers initiative and other sources. 2. Similarly, in the Discussion (p.29) it is stated that [this approach] ‘… will allow countries to cost and integrate programmes …’ This may only happen if these programmes are demonstrated to be cost-effective when compared with other interventions such as universal testing and treatment. Other obstacles remain, such as moral and religious objections to the removal of legal obstacles, however these too can be addressed with multiple strategies involving advocacy, education, and dialogue. It is not suggested that the paper should discuss these interventions here - only the cost-effectiveness issue, because that is directly germane to the arguments made by the authors. | Thank you for this suggestion. We agree that adding a short discussion of the difference between effectiveness and cost-effectiveness in evaluating HIV intervention would improve the manuscript.  We have made the following clarifications and additions in response:  On pg. 6, lines 162-164 we clarified what we mean by “optimizing the effectiveness of core HIV programmes” by adding the following phrase at the end of the sentence:  “The process to re-envision the enablers began with an in-house review at UNAIDS (led by JAI) of current understanding of how the enablers, especially the societal enablers, optimize the effectiveness of core HIV programmes (e.g. lead to increases in uptake of HIV testing, initiation of treatment, adherence to treatment, etc.).”  With regard to cost-effectiveness, we have added the following text at the end of the first paragraph of the Discussion on pg. 28, lines 462-464:  “Informed by this process, the 3 S’s framework, the three top-line and 15 detailed evidence-based targets, and the 15 indicators for assessing progress towards these targets, will support countries to refine program priorities, track progress, and measure the programme- and cost-effectiveness of societal enabling approaches for integration into their HIV responses”.  We have also added the following limitation on pg. 29, lines 514-522:  “Second, none of the studies reviewed assessed the cost or cost-effectiveness of the societal enabling approaches evaluated, which may slow adoption of these approaches at the country-level. While costing and cost-effectiveness research exists for HIV interventions and social and behaviour change programs, there is a dearth of evidence that specifically examines the cost-effectiveness of approaches that address societal enablers for HIV outcomes. Cost-effectiveness analysis compares the cost per unit outcome (e.g. new HIV diagnosis, new treatment initiation, new client virally suppressed, etc.) between two or more programmes [66]. Such data would be especially helpful given the large number of societal enabling approaches that have been piloted and found to positively influence the effectiveness of HIV services. Research is urgently needed to address this gap.” |
| The abstract states that ‘…no global targets exist to spur funding and action…’ As the authors will be aware, in June 2021 the UN General Assembly adopted the 2021 Political Declaration on HIV/AIDS, which contains relevant commitments and targets. The article should now be updated to reflect these developments otherwise it will be out of date and misleading. | Thank you for noting this. We had submitted the paper prior to the high-level meeting but agree that the text should now be updated throughout to reflect the outcomes of the high-level meeting. We have done so throughout the abstract, at the end of the introduction, and in the results sub-section on proposed targets for monitoring progress on societal enablers or lifting impediments. All edits made are noted in the tracked changes version of the revised manuscript we uploaded. |
| Line 212: ‘Inequitable gender norms were associated with more HIV testing…’ Should this be ‘less HIV testing’? Compare with line 215 ‘Community mobilization interventions to reduce IPV led to increased HIV testing…’ If correct, this may need explanation. | Thank you for noting this. We have clarified our summary of the results of this study in the results section on pg. 18-19, lines 297-311:  “ A study in South Africa that examined the influence of inequitable gender norms on HIV service use behaviours found that both women and men living with HIV who endorsed inequitable gender norms were less likely to be currently taking antiretrovirals, (i.e., women who endorsed norms accepting men’s control over and violence towards women; men as the main / sole decision-maker in a couple; and men as reluctant to seek care/help during illness; and men who endorsed norms around men as the main/sole decision maker in a couple). This study also found that receiving an HIV test in the past year was significantly associated with endorsement of inequitable gender norms (among women only, and especially for norms suggesting women have the primary/exclusive responsibility as family caretaker). While unexpected, additional analyses conducted by the study authors suggested that the association was likely due to the greater likelihood of testing after having children/during pregnancy, as HIV testing is routinely offered at antenatal services in South Africa, and as women with children were more likely to endorse those primary caretaker norms.” We also updated the data reported in Table 1 for this study to reflect the additional data reported/summarized in the paper. See tracked changes on pg. 12. |
